# Unpacking the Role of Financial Incentives in the Motivation and Individual Performance of Nurses in LMICs: A Scoping Review Protocol

**DOI:** 10.1002/puh2.70404

**Published:** 2026-10-11

**Authors:** Abdellah Selmi, Diallo Djibrine, Zakaria Belrhiti

**Affiliations:** ^1^ Mohammed VI International School of Public Health Mohammed VI University of Health Sciences (UM6SS) Casablanca Morocco; ^2^ Public Health & Management Department Mohammed VI Center for Research and Innovation (CM6RI) Rabat Morocco

**Keywords:** financial incentives, job performance, low‐ and middle‐income country (LMIC), motivation, nurses, scoping review

## Abstract

**Background:**

Health systems in low‐ and middle‐income countries (LMICs) continue to face persistent challenges related to health worker performance and quality of care, exacerbated by a global shortage of nurses. Financial incentive strategies (FIS), which include performance‐based financing, bonuses and allowances, are extensively implemented as mechanisms to enhance the motivation and individual performance of health workers, including nurses. Nevertheless, the effects of these strategies exhibit considerable variability and are highly contingent upon specific contextual factors.

**Objective:**

We aim to synthesise evidence on how FIS are designed and implemented in LMICs to examine how nurse motivation and individual performance are defined and measured and to identify the underlying theoretical assumptions through which FIS may influence nurses’ motivation and individual performance.

**Methods:**

We adopted the scoping review methodology following guidance from Arksey and O'Malley and the Joanna Briggs Institute and PRISMA‐ScR reporting standards. The search will cover studies published from 2000 onwards in PubMed, Scopus, Web of Science and PsycINFO, with additional grey literature. Two reviewers will independently screen studies and extract data, with discrepancies resolved by a third reviewer. Data will be charted using a standardised form. Findings will be synthesised through narrative analysis.

**Results:**

This review will provide a comprehensive overview of FIS in LMICs, including their design and implementation features, whilst focussing on the underlying mechanisms by which FIS interventions improve the motivation and performance of nurses in LMICs.

**Discussion:**

This scoping review aims to map the existing evidence in LMICs on FIS‐targeting nurses, its aspects and their relationship with motivation and individual performance and explores the theoretical assumptions of this relationship.

**Conclusion:**

The findings are expected to address a significant evidence gap concerning nurse‐specific workforce interventions in LMICs and to inform ongoing health policy reforms aimed at improving and sustaining motivation, retention and care delivery.

## Graphical abstract

This scoping review protocol outlines a comprehensive strategy to map how financial incentive strategies (FIS) influence nurses’ motivation and individual performance in low‐ and middle‐income countries (LMICs). Grounded in agency, expectancy, self‐determination and psychological contract theories, the synthesis examines FIS design components and measurement frameworks to provide key insights for ongoing health workforce policy reforms.

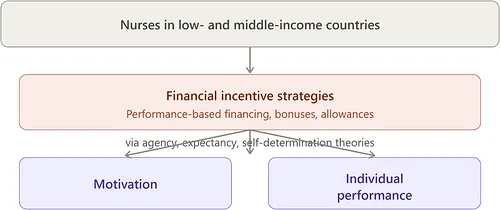

## 1 Introduction

Health systems, in low‐ and middle‐income countries (LMICs), are continually challenged by issues related to poor quality of care and limited motivation and the performance of health workers, leading to a non‐utilisation of health services, causing 8.6 million annual deaths amenable to healthcare [1]. Among these challenges, the global shortage of healthcare personnel, particularly nurses, exacerbates these problems, resulting in missed care, increased health disparities and heightened risks to patients safety and mortality [2, 3]. To effectively address these persistent challenges, numerous LMICs have implemented financial incentive strategies (FIS), such as performance‐based bonuses and allowances, aiming to enhance workforce motivation and improve healthcare outcomes [4]. In LMICs, nurses’ motivation and individual performance are constrained by a constellation of interrelated organisational and structural factors [5]. These include poor working conditions [6], the inequitable allocation of equipment and resources [7], low remuneration [8], excessive workloads and perceived injustice in the distribution of financial incentives [9]. Compounding, these challenges are difficulties in achieving work‐life balance [10] and the prevalence of transactional leadership styles that offer limited support for nurses’ motivation [11]. Collectively, these factors contribute to burnout and absenteeism among nursing staff [12], thereby undermining the overall performance and sustainability of health systems in resource‐constrained settings. Compensation for nurses can be conceptually divided into two distinct components: basic remuneration and financial incentives. Basic remuneration encompasses the total reward package, including basic pay associated with job role, position or time worked [13]. Financial incentives are direct monetary payments [14], incentivising specific behaviours to drive performance, improve quality of care and enhance delivery efficiency [15].

According to Herzberg's theory, basic pay is considered a hygiene factor along with job security and working conditions, which prevent dissatisfaction but do not inherently motivate employees [16]. In contrast, financial incentives utilise targeted instruments such as bonuses and allowances to enhance individual performance. These encompass accommodation, overtime and shift allowances, alongside rural or district posting differentials, all of which aim to attract and retain healthcare professionals [17, 18]. As Buchan and colleagues state, an incentive is a particular form of payment intended to achieve a specific change in behaviour [19].

### 1.1 FIS Are Complex Social Systems

FIS are considered complex adaptive systems as they are highly dependent on contextual conditions, such as high workloads and staff shortages [20], and are often characterised by non‐linearity, which can result in both positive and negative outcomes [21]. Positive outcomes include a greater sense of achievement, professional recognition [22, 23], improved work engagement [24] and increased self‐reflection [13]. Negative outcomes can include perceived unfairness, goal displacement, deviant behaviours [25], interpersonal tensions and stress [26]. FIS are also connected to career development and perceived recognition over money [27, 28].

FIS comprise several interacting components, with interventions tailored to different LMIC contexts and stakeholders varying in design and in the actors involved. FIS encompass more than a single financial tool [29]. FIS include direct forms such as allowances, hazard pay for high‐risk environments, hardship supplements and location‐based incentives, all of which facilitate the recruitment and retention of staff in rural and underserved areas [17]. Another form of FIS is a component of the Performance‐Based Financing (PBF) programme, in which financial incentives are conditional on predefined performance indicators related to the quantity and quality of health services [30]. There are also indirect forms of FIS, which consist of support for daily living costs and easing work‐life balance pressures, such as meal subsidies, clothing allowances, housing support, transportation coverage and childcare subsidies, including on‐site crèches [19].

FIS design involves a complex set of decisions about what to measure, who to reward and how to structure payments to effectively influence health workers’ behaviour [31].

Key design considerations include payment frequency, the amount, the time lag between reporting and payment and the inclusion of additional features. FIS can integrate various verification processes, such as routine monitoring, external audits and community‐based verification [30], drawing on both quantitative indicators (such as service delivery volumes, adherence to clinical protocols and absenteeism) and qualitative indicators (such as structured checklists and ex ante and ex post verification) [32].

Evidence shows that FIS can improve nurses’ individual performance by clarifying goals and reinforcing clinical quality [33]. These improvements could lead to better healthcare access, higher standards and greater equity in service delivery [32].

However, when implemented in real‐world settings, FIS face several challenges limiting their effect on health workers’ performance [34]. These challenges include a lack of trust in management, delayed payments and a mismatch between the total amount of incentives and nurses’ expectations [35], crowding out of intrinsic motivation [36], gaming and data falsification [37] and finally goal displacement, understood as the prioritisation of tasks associated with incentivised activities, often at the expense of essential non‐incentivised care [38].

### 1.2 Underlying Motivational Theories

Given the centrality of motivation to these challenges, it is necessary to clarify how this concept is defined and how it relates to individual performance. Motivation is understood here as the dynamic process encompassing the initiation, direction, intensity and a persistence of behaviour necessary to perform functions and achieve objectives [39]. Individual performance, by contrast, is defined as the combination of personal skills and the motivation to deploy them effectively within a given context [40]. Both elements are important and interconnected; motivation enhances job performance, strengthens effort and perseverance in the face of adversity and motivates individuals to set and pursue ambitious goals [41].

Several theories can be applied: agency theory, the expectancy theory of motivation, self‐determination theory and psychological contract theory [42].

The agency theory holds that incentives align the interests of health workers with system objectives by linking pay to performance [43]. The expectancy theory explains how incentives influence effort through expectations, and the value of rewards [44].

Self‐Determination Theory emphasises that financial incentives can either support or undermine motivation, depending on whether they are perceived as informational or controlling [45]. Psychological contract theory highlights how remuneration systems influence implicit expectations between health workers and organisations [46] (see Figure 1 generated by Gemini).

**FIGURE 1:**
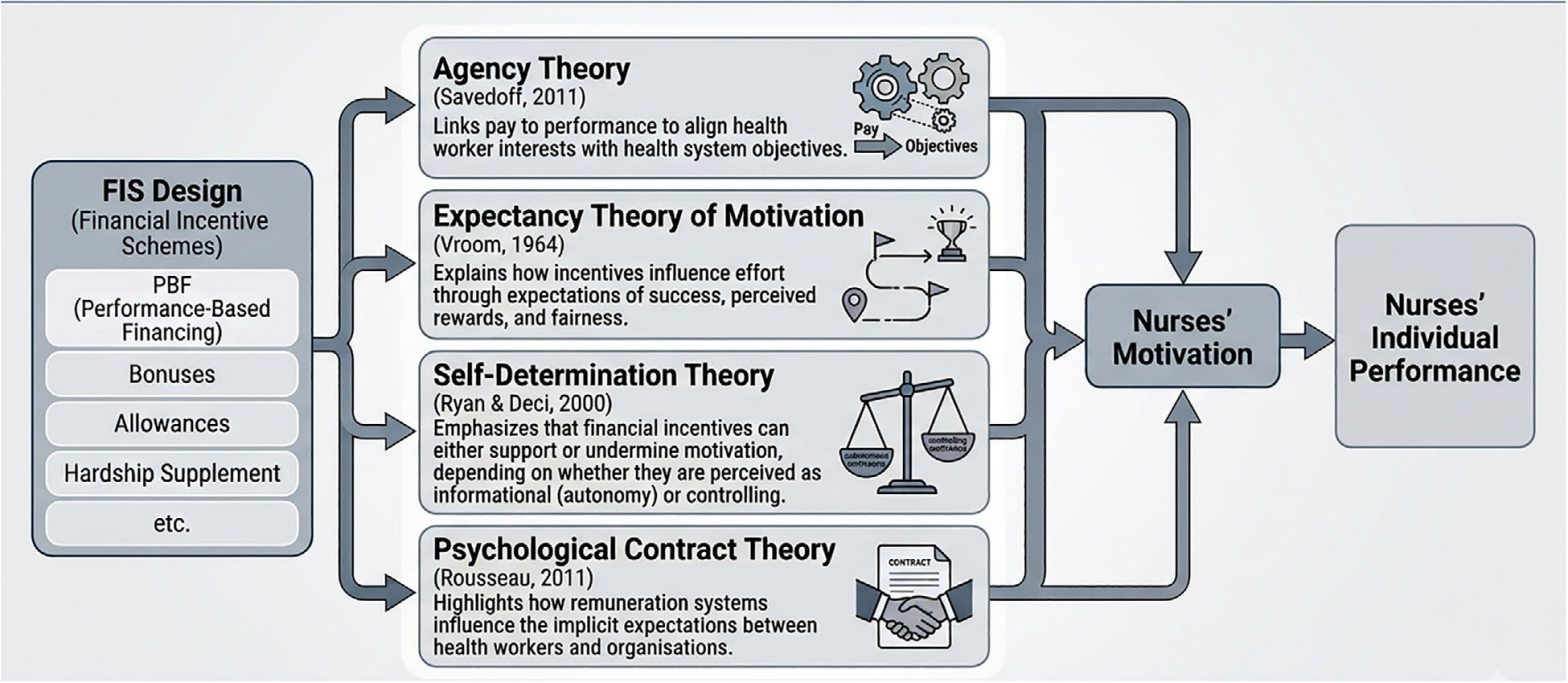
A conceptual framework of how FIS underlies nurse motivation and individual performance.

Little research has examined the complex relationships among FIS, motivation and performance among nurses in LMICs, as well as the intricate relationships between contextual factors and other intervention components, including supervision, infrastructure and training [1, 2, 47]. Most studies about FIS have traditionally focused on physicians with limited attention to nurses [48]. Few studies have been carried out in the Middle East and North Africa (MENA) region [5]. To address this gap, this scoping review set out to synthesise available evidence on how FIS influence nurses' motivation and individual performance in LMICs. It will provide practical and policy lessons learnt that could be used to adapt existing FIS in LMICs and in the MENA region.

## 2 Methods

We will follow Arksey and O'Malley [49] and the JBI methodological guidance for scoping reviews [50], and the PRISMA‐ScR reporting guidelines [51]. We will systematically identify and map relevant studies to provide a comprehensive overview of the FIS's effect on nurse motivation and individual performance across health systems in LMICs. This process will involve five steps: (1) refining the review question; (2) identifying relevant studies; (3) selecting studies; (4) charting the data; and (5) collating, summarising and reporting the results [49].

### 2.1 Identifying the Research Question

This scoping review aims to answer the following questions:

- *How are FIS designed and implemented in LMICs?*
- *How are the motivation and individual performance of nurses defined and measured?*
- *What are the underlying theoretical assumptions of the relationships between FIS, nurses’ motivation and individual performance in LMICs?*

### 2.2 Identifying Relevant Studies

The search will include all primary study types published in Arabic, French or English since the 2000s. Since 2000, PBF has been widely promoted in LMICs [52]. Literature reviews, including meta‐analyses, will be excluded to avoid duplicating primary data.

We will search four databases: Scopus, Web of Science and PubMed for the comprehensive scope in health services research, and the PsycINFO database will be included for its emphasis on psychological research.

The grey literature search will also be conducted. Single‐concept searches will be employed, and results will be manually screened using the components of the SPICE framework (Table 2), without date restrictions, relevant literature will be retrieved from preprint and thesis databases, Management Sciences for Health (MSH), the World Bank Open Knowledge Repository (OKR), the WHO Institutional Repository for Information Sharing (IRIS) and the International Initiative for Impact Evaluation (3ie), Google Scholar and Google.

We developed our search strategy using the conceptual framework (SPICE) (see Tables 1 and 2). We also used MeSH terms (see Tables 1 and 2 and Supporting Information file 1 included search strategy).

**TABLE 1:** The strategy using the conceptual framework (SPICE) framework.

Element	Definition
Settings	LMICs as described by the World Bank
Perspective	Nurses
Intervention	Financial incentive strategies (PBF bonuses, allowance etc.)
Comparison	Not appropriate
Evaluation	Motivation, and/or individual performance

Abbreviations: LMICs, low‐ and middle‐income countries; PBF, performance‐based financing.

**TABLE 2:** Search terms.

Nurses	nurs*, ‘nursing staff’, ‘nursing personnel’, ‘health personnel’, ‘healthcare professional*’, ‘health worker*’, ‘healthcare worker*’
Financial incentives	‘financial incentive*’, ‘monetary incentive*’, ‘payment incentive*’, ‘pay for performance’, ‘performance‐based pay’, remuneration, compensation, bonus*, ‘performance‐based reimbursement’, ‘quality‐based payment*’, among others
Motivation	motivat*, ‘employee motivation’, ‘extrinsic motivation’, ‘intrinsic motivation’, satisfaction, ‘employee engagement’, ‘job satisfaction’
Individual performance	perform*, ‘job performance’, ‘work performance’, productivity, ‘task performance’
Geographic scope	‘low‐income countr*’, ‘middle‐income countr*’, ‘LMIC*’, ‘developing countr*’ or specific country names

The protocol has been registered with the Open Science Framework (OSF) on 24 March 2026 (Registration DOI: 10.17605/OSF.IO/XW6J9).

### 2.3 Study Selection

To ensure consistency and rigour in the selection process, both primary studies and grey literature will be included. We will use a two‐step process with the Rayyan web application:

Title and Abstract Screening: Two independent reviewers, A.S. and D.D., will screen titles and abstracts against the inclusion criteria (see Table 3). Discrepancies will be resolved by the third reviewer, Z.B.

**TABLE 3:** Inclusion and exclusion criteria.

	Inclusion criteria	Exclusion criteria
**Settings**	Studies conducted in countries classified as LMIC by the World Bank at the time of the study or publication	Studies from high‐income countries
**Population**	Studies focusing on nurses, or studies where nurses are a subgroup within a broader group of health professionals	Studies not including nurses
**Intervention**	Any form of FIS (e.g., performance‐based financing, bonuses, salary supplements, hardship allowances) and its relationship with nurse motivation and/or individual performance	Studies including only non‐financial incentives
**Language**	Arabic, French and English	Other languages
**Time period covered**	From 2000 to current date	Out‐of‐period studies

Abbreviations: FIS, financial incentive strategies; LMICs, low‐ and middle‐income countries.

Full‐Text Review: A.S. and D.D. will assess the full text of relevant articles and grey literature against the inclusion criteria. Reasons for exclusion will be recorded. Grey literature will be excluded if it lacks primary data, duplicates findings from a study already included or provides insufficient detail on FIS design, motivation and/or individual performance.

### 2.4 Data Charting

A standardised data charting form will be developed and piloted across five studies to address the scoping review's research question. The form will subsequently be refined on the basis of the pilot results. All revisions will be documented transparently in the final review.

During the data extraction process, two reviewers (A.S. and D.D.) will independently extract data from the included studies (see Supporting Information file 2) and from grey literature sources (see Supporting Information file 3). On a random sample of 20% of included studies, we will measure the inter‐rater agreement coefficient to ensure accuracy and consistency.

We did not conduct a methodological quality appraisal of the included literature, as critical appraisal is optional in scoping reviews under the PRISMA‐ScR framework. This review aims to provide a comprehensive map of the available literature, regardless of study quality, to identify gaps and visualise the broader evidence landscape.

### 2.5 Collating, Summarising and Reporting the Results

The extracted data will be synthesised to provide a descriptive overview of the literature, using both numerical and qualitative methods consistent with scoping review methodology. We will also use narrative analysis to synthesise the FIS design in relation to motivation and individual performance.

We will report the results according to the PRISMA Extension for Scoping Reviews guidelines [51] (Supporting Information file S4: PRISMA checklist).

#### 2.5.1 Textual Narrative Synthesis

It is a structured, descriptive approach to synthesising evidence, particularly well‐suited to scoping reviews that include both quantitative and qualitative studies [53] (Supporting Information file 5: textual narrative synthesis). To ensure accuracy and consistency, two reviewers (A.S. and D.D.) will independently conduct each step of the synthesis; discrepancies will be resolved through discussion, with arbitration by a third reviewer (Z.B.) where consensus cannot be reached.

##### 2.5.1.1 Defining Sub‐Groups and Studies

This step arranges disparate study types into more homogeneous sub‐groups, which aids in the synthesis of different types of evidence; for this scoping review, studies will be sorted by FIS design.

##### 2.5.1.2 Providing Narrative Commentary by Study

For each study within a subgroup, we will write a short narrative commentary to address the FIS's influence on nurses’ motivation and individual performance, as well as the underlying theoretical assumptions.

##### 2.5.1.3 Synthesising Narrative Commentary by Sub‐Group

The individual narrative commentaries for studies within the same subgroup will be synthesised to describe key findings, similarities and differences for each FIS design (e.g., PBF, allowances). We will highlight the strengths of the evidence and then provide a summary conclusion.

##### 2.5.1.4 Synthesising Overall Findings and Identifying Gaps

In the final step, we will compare the results of the subgroup synthesis to draw broader conclusions. We will also identify the strengths and limitations of the evidence and highlight gaps in the existing literature.

## 3 Discussion

This scoping review aims to examine how FIS are designed and implemented in LMICs, how nurses’ motivation and individual performance are defined and measured, and the theoretical assumptions underlying these strategies. By synthesising primary studies and grey literature, the review is expected to identify key design features, bundled interventions and evidence gaps. The planned dual independent screening, standardised charting and textual narrative synthesis will enhance transparency and reproducibility.

Grounded in agency and expectancy theories, FIS can align nursing practices with broader health system objectives. Nevertheless, these designs often prove ineffective when resource shortages, inadequate infrastructure or delayed payments hinder the translation of effort into improved performance. Self‐Determination Theory suggests that rigid or controlling incentives may diminish nurses’ intrinsic motivation and professional pride through a crowding‐out effect [45]. Perceived procedural and distributive inequities, including non‐transparent verification processes or unequal payments relative to physicians, frequently lead to breaches of the psychological contract, interpersonal conflict and opportunistic behaviours such as data falsification and goal displacement [54, 55]. Additionally, nurses face substantial structural barriers, excessive workloads and hierarchical management structures [26, 56].

Therefore, financial incentives cannot replace adequate base salaries, safe working conditions or essential operational resources [57, 58].

## 4 Limitations

As this work is part of a PhD study, we will balance comprehensiveness with what is feasible and timely. No quality appraisal will be done because the goal of this scoping review is to map and describe the FIS, rather than to evaluate their efficacy [51]. Some studies that group nurses with other health workers make it hard to extract nurse‐specific findings, but it is worth noting that our extraction form includes other recipients beyond the nurses’ category. This protocol lays the groundwork for future research that combines various methods and tracks the long‐term effects of FIS on nurses worldwide.

## 5 Conclusion

This scoping review will address a timely and important gap in evidence on FIS for nurses in LMICs. It will also be highly relevant to the current health reform in Morocco. This review will provide new insights into the framing of FIS designs, the contexts in which they operate, and the measures of motivation, and individual performance used in LMICs. The findings will serve as the starting point for qualitative research.

## Author Contributions

**Abdellah Selmi**: conceptualisation, methodology, software, data curation, investigation, validation, formal analysis, visualisation, project administration, writing – original draft, writing – review and editing, resources. **Diallo Djibrine**: data curation, resources, software, methodology, validation, formal analysis, writing – review and editing. **Zakaria Belrhiti**: conceptualisation, methodology, validation, supervision.

## Funding

These authors have nothing to report.

## Ethics Statement

This study is based solely on literature reviews and reports.

## Conflicts of Interest

The authors declare no conflicts of interest.

## Supporting information




**Supporting Information file 1**: puh270404‐sup‐0001‐SuppMat.docx

**Supporting Information file 2**: puh270404‐sup‐0002‐SuppMat.docx

**Supporting Information file 3**: puh270404‐sup‐0003‐SuppMat.docx

**Supporting Information file 4**: puh270404‐sup‐0004‐SuppMat.docx

**Supporting Information file 5**: puh270404‐sup‐0005‐SuppMat.docx

## Data Availability

All data used in this study are available from the corresponding author upon request.
