# Supplementary material for: Unpacking the Role of Financial Incentives in the Motivation and Individual Performance of Nurses in LMICs: A Scoping Review Protocol
Source: Public Health Chall. 2026 Oct 11;5(4):e70404. doi: 10.1002/puh2.70404 (PMC13654303; doi:10.1002/puh2.70404)
Supplement: Supplementary file 1 [file PUH2-5-e70404-s001.docx]

| Supplementary file 1: Research strategy | |
| --- | --- |
| Search engine | Search terms |
| PsychInfo  Date 2000-now | (TI OR AB OR SU OR DE) (  nurs* OR "nursing staff" OR "nursing personnel"  )  AND  (TI OR AB OR SU OR DE) (  "health personnel" OR "healthcare professional*" OR "health worker*" OR "healthcare worker*" OR "medical staff"  )  AND  (TI OR AB OR SU OR DE) (  "financial incentive*" OR "monetary incentive*" OR "payment incentive*" OR "pay for performance"  OR "performance-based pay" OR remuneration OR compensation OR bonus*  OR (pay* N3 performance) OR (pay* N3 quality)  OR ((incentive* OR compensatory OR reimbursement) N1 plan*)  OR "contract service" OR "result based financing" OR "performance based financing"  OR ((performance OR output OR result*) N2 (finance* OR pay* OR disburs* OR incentive* OR contract*))  OR "economic incentive*" OR "reimbursement incentive*"  OR ((financial OR economic) N1 (reward* OR bonus*))  OR "pay for value" OR "performance-based reimbursement"  OR ((doctor* OR physician* OR nurse* OR practitioner* OR provider*) N4 (bonus* OR incentive* OR financial OR monetary OR payment OR pay OR salary))  OR "incentive program" OR "quality-based payment*" OR "quality incentive*" OR "output-based payment"  )  AND  (TI OR AB OR SU OR DE) (  motivat* OR "employee motivation" OR "extrinsic motivation" OR "intrinsic motivation"  OR trust OR "employee attitudes" OR satisfaction OR "career change"  OR "employee engagement" OR "employee retention" OR "job enrichment" OR "job involvement"  OR "organizational commitment" OR "quality of work life" OR "role satisfaction"  OR "job satisfaction" OR relationship OR teamwork OR retention  )  AND  (TI OR AB OR SU OR DE) (  perform* OR "job performance" OR "work performance" OR productivity  OR "contextual performance" OR "task performance"  )  AND  (TI OR AB OR SU OR DE) (  "low-income countr*" OR "middle-income countr*" OR LMIC* OR "developing countr*" OR "resource-constrained"  OR Africa OR Asia  OR (AFGHANISTAN OR ALBANIA OR ALGERIA OR ANGOLA OR ARGENTINA OR ARMENIA OR BANGLADESH  OR BENIN OR BHUTAN OR BOLIVIA OR BOTSWANA OR BRAZIL OR "BURKINA FASO" OR BURUNDI OR CAMBODIA  OR CAMEROON OR "CENTRAL AFRICAN REPUBLIC" OR CHAD OR CHINA OR COLOMBIA OR COMOROS OR CONGO  OR "COTE D'IVOIRE" OR CUBA OR DJIBOUTI OR "DOMINICAN REPUBLIC" OR ECUADOR OR EGYPT OR "EL SALVADOR"  OR ETHIOPIA OR FIJI OR GABON OR GAMBIA OR GHANA OR GUATEMALA OR GUINEA OR HAITI OR HONDURAS  OR INDIA OR INDONESIA OR IRAN OR IRAQ OR JAMAICA OR JORDAN OR KAZAKHSTAN OR KENYA OR KIRIBATI  OR KYRGYZSTAN OR LAOS OR LEBANON OR LESOTHO OR LIBERIA OR MADAGASCAR OR MALAWI OR MALAYSIA  OR MALDIVES OR MALI OR MAURITANIA OR MAURITIUS OR MEXICO OR MOLDOVA OR MONGOLIA OR MOROCCO  OR MOZAMBIQUE OR MYANMAR OR NAMIBIA OR NEPAL OR NICARAGUA OR NIGER OR NIGERIA OR PAKISTAN  OR "PAPUA NEW GUINEA" OR PARAGUAY OR PERU OR PHILIPPINES OR RWANDA OR "SAO TOME" OR SENEGAL  OR "SIERRA LEONE" OR "SOLOMON ISLANDS" OR SOMALIA OR "SOUTH AFRICA" OR "SOUTH SUDAN" OR "SRI LANKA"  OR SUDAN OR SURINAME OR SYRIA OR TAJIKISTAN OR TANZANIA OR THAILAND OR "TIMOR-LESTE"  OR TOGO OR TUNISIA OR TURKEY OR UGANDA OR UKRAINE OR UZBEKISTAN OR VANUATU OR VENEZUELA  OR VIETNAM OR YEMEN OR ZAMBIA OR ZIMBABWE)  ) |
| PubMed  Date 2000-now | (nurs* OR "nursing staff" OR "nursing personnel"[tiab])  AND  ("health personnel" OR "healthcare professional*" OR "health worker*" OR "healthcare worker*" OR "medical staff"[tiab])  AND  ("financial incentive*" OR "monetary incentive*" OR "payment incentive*" OR "pay for performance"  OR "performance-based pay" OR remuneration OR compensation OR bonus*  OR "pay performance" OR "pay quality"  OR "incentive plan*" OR "compensatory plan*" OR "reimbursement plan*"  OR "contract service" OR "result based financing" OR "performance based financing"  OR "performance finance*" OR "output finance*" OR "output pay*" OR "result* finance*" OR "result* incentive*"  OR "economic incentive*" OR "reimbursement incentive*"  OR "financial reward*" OR "financial bonus*" OR "economic reward*" OR "economic bonus*"  OR "pay for value" OR "performance-based reimbursement"  OR "doctor* bonus*" OR "doctor* incentive*" OR "physician* bonus*" OR "physician* incentive*"  OR "nurse* bonus*" OR "nurse* incentive*" OR "practitioner* bonus*" OR "provider* bonus*"  OR "doctor* payment*" OR "physician* payment*" OR "nurse* payment*"  OR "incentive program*" OR "quality-based payment*" OR "quality incentive*" OR "output-based payment"[tiab])  AND  (motivat* OR "employee motivation" OR "extrinsic motivation" OR "intrinsic motivation"  OR trust OR "employee attitude*" OR satisfaction OR "career change"  OR "employee engagement" OR "employee retention" OR "job enrichment" OR "job involvement"  OR "organizational commitment" OR "quality of work life" OR "role satisfaction"  OR "job satisfaction" OR relationship* OR teamwork OR retention[tiab])  AND  (perform* OR "job performance" OR "work performance" OR productivity  OR "contextual performance" OR "task performance"[tiab])  AND  ("low-income countr*" OR "middle-income countr*" OR LMIC* OR "developing countr*" OR "resource-constrained"  OR Africa OR Asia  OR (AFGHANISTAN OR ALBANIA OR ALGERIA OR ANGOLA OR ARGENTINA OR ARMENIA OR BANGLADESH  OR BENIN OR BHUTAN OR BOLIVIA OR BOTSWANA OR BRAZIL OR "BURKINA FASO" OR BURUNDI OR CAMBODIA  OR CAMEROON OR "CENTRAL AFRICAN REPUBLIC" OR CHAD OR CHINA OR COLOMBIA OR COMOROS OR CONGO  OR "COTE D'IVOIRE" OR CUBA OR DJIBOUTI OR "DOMINICAN REPUBLIC" OR ECUADOR OR EGYPT OR "EL SALVADOR"  OR ETHIOPIA OR FIJI OR GABON OR GAMBIA OR GHANA OR GUATEMALA OR GUINEA OR HAITI OR HONDURAS  OR INDIA OR INDONESIA OR IRAN OR IRAQ OR JAMAICA OR JORDAN OR KAZAKHSTAN OR KENYA OR KIRIBATI  OR KYRGYZSTAN OR LAOS OR LEBANON OR LESOTHO OR LIBERIA OR MADAGASCAR OR MALAWI OR MALAYSIA  OR MALDIVES OR MALI OR MAURITANIA OR MAURITIUS OR MEXICO OR MOLDOVA OR MONGOLIA OR MOROCCO  OR MOZAMBIQUE OR MYANMAR OR NAMIBIA OR NEPAL OR NICARAGUA OR NIGER OR NIGERIA OR PAKISTAN  OR "PAPUA NEW GUINEA" OR PARAGUAY OR PERU OR PHILIPPINES OR RWANDA OR "SAO TOME" OR SENEGAL  OR "SIERRA LEONE" OR "SOLOMON ISLANDS" OR SOMALIA OR "SOUTH AFRICA" OR "SOUTH SUDAN" OR "SRI LANKA"  OR SUDAN OR SURINAME OR SYRIA OR TAJIKISTAN OR TANZANIA OR THAILAND OR "TIMOR-LESTE"  OR TOGO OR TUNISIA OR TURKEY OR UGANDA OR UKRAINE OR UZBEKISTAN OR VANUATU OR VENEZUELA  OR VIETNAM OR YEMEN OR ZAMBIA OR ZIMBABWE[tiab]) |
| Web of Sciences  Date 2000-now | TS=(nurs* OR "nursing staff" OR "nursing personnel")  AND  TS=("health personnel" OR "healthcare professional*" OR "health worker*" OR "healthcare worker*" OR "medical staff")  AND  TS=("financial incentive*" OR "monetary incentive*" OR "payment incentive*" OR "pay for performance"  OR "performance-based pay" OR remuneration OR compensation OR bonus*  OR (pay* NEAR/3 performance) OR (pay* NEAR/3 quality)  OR ("incentive plan*" OR "compensatory plan*" OR "reimbursement plan*")  OR "contract service" OR "result based financing" OR "performance based financing"  OR ((performance OR output OR result*) NEAR/2 (finance* OR pay* OR disburs* OR incentive* OR contract*))  OR "economic incentive*" OR "reimbursement incentive*"  OR ((financial OR economic) NEAR/1 (reward* OR bonus*))  OR "pay for value" OR "performance-based reimbursement"  OR ((doctor* OR physician* OR nurse* OR practitioner* OR provider*) NEAR/4 (bonus* OR incentive* OR financial OR monetary OR payment OR pay OR salary))  OR "incentive program*" OR "quality-based payment*" OR "quality incentive*" OR "output-based payment")  AND  TS=(motivat* OR "employee motivation" OR "extrinsic motivation" OR "intrinsic motivation"  OR trust OR "employee attitude*" OR satisfaction OR "career change"  OR "employee engagement" OR "employee retention" OR "job enrichment" OR "job involvement"  OR "organizational commitment" OR "quality of work life" OR "role satisfaction"  OR "job satisfaction" OR relationship* OR teamwork OR retention)  AND  TS=(perform* OR "job performance" OR "work performance" OR productivity  OR "contextual performance" OR "task performance")  AND  TS=("low-income countr*" OR "middle-income countr*" OR LMIC* OR "developing countr*" OR "resource-constrained"  OR Africa OR Asia  OR (AFGHANISTAN OR ALBANIA OR ALGERIA OR ANGOLA OR ARGENTINA OR ARMENIA OR BANGLADESH  OR BENIN OR BHUTAN OR BOLIVIA OR BOTSWANA OR BRAZIL OR "BURKINA FASO" OR BURUNDI OR CAMBODIA  OR CAMEROON OR "CENTRAL AFRICAN REPUBLIC" OR CHAD OR CHINA OR COLOMBIA OR COMOROS OR CONGO  OR "COTE D'IVOIRE" OR CUBA OR DJIBOUTI OR "DOMINICAN REPUBLIC" OR ECUADOR OR EGYPT OR "EL SALVADOR"  OR ETHIOPIA OR FIJI OR GABON OR GAMBIA OR GHANA OR GUATEMALA OR GUINEA OR HAITI OR HONDURAS  OR INDIA OR INDONESIA OR IRAN OR IRAQ OR JAMAICA OR JORDAN OR KAZAKHSTAN OR KENYA OR KIRIBATI  OR KYRGYZSTAN OR LAOS OR LEBANON OR LESOTHO OR LIBERIA OR MADAGASCAR OR MALAWI OR MALAYSIA  OR MALDIVES OR MALI OR MAURITANIA OR MAURITIUS OR MEXICO OR MOLDOVA OR MONGOLIA OR MOROCCO  OR MOZAMBIQUE OR MYANMAR OR NAMIBIA OR NEPAL OR NICARAGUA OR NIGER OR NIGERIA OR PAKISTAN  OR "PAPUA NEW GUINEA" OR PARAGUAY OR PERU OR PHILIPPINES OR RWANDA OR "SAO TOME" OR SENEGAL  OR "SIERRA LEONE" OR "SOLOMON ISLANDS" OR SOMALIA OR "SOUTH AFRICA" OR "SOUTH SUDAN" OR "SRI LANKA"  OR SUDAN OR SURINAME OR SYRIA OR TAJIKISTAN OR TANZANIA OR THAILAND OR "TIMOR-LESTE"  OR TOGO OR TUNISIA OR TURKEY OR UGANDA OR UKRAINE OR UZBEKISTAN OR VANUATU OR VENEZUELA  OR VIETNAM OR YEMEN OR ZAMBIA OR ZIMBABWE)) |
| Scopus  Date 2000-now | TITLE-ABS-KEY(nurs* OR "nursing staff" OR "nursing personnel")  AND  TITLE-ABS-KEY("health personnel" OR "healthcare professional*" OR "health worker*" OR "healthcare worker*" OR "medical staff")  AND  TITLE-ABS-KEY("financial incentive*" OR "monetary incentive*" OR "payment incentive*" OR "pay for performance"  OR "performance-based pay" OR remuneration OR compensation OR bonus*  OR (pay* W/3 performance) OR (pay* W/3 quality)  OR ("incentive plan*" OR "compensatory plan*" OR "reimbursement plan*")  OR "contract service" OR "result based financing" OR "performance based financing"  OR ((performance OR output OR result*) W/2 (finance* OR pay* OR disburs* OR incentive* OR contract*))  OR "economic incentive*" OR "reimbursement incentive*"  OR ((financial OR economic) W/1 (reward* OR bonus*))  OR "pay for value" OR "performance-based reimbursement"  OR ((doctor* OR physician* OR nurse* OR practitioner* OR provider*) W/4 (bonus* OR incentive* OR financial OR monetary OR payment OR pay OR salary))  OR "incentive program*" OR "quality-based payment*" OR "quality incentive*" OR "output-based payment")  AND  TITLE-ABS-KEY(motivat* OR "employee motivation" OR "extrinsic motivation" OR "intrinsic motivation"  OR trust OR "employee attitude*" OR satisfaction OR "career change"  OR "employee engagement" OR "employee retention" OR "job enrichment" OR "job involvement"  OR "organizational commitment" OR "quality of work life" OR "role satisfaction"  OR "job satisfaction" OR relationship* OR teamwork OR retention)  AND  TITLE-ABS-KEY(perform* OR "job performance" OR "work performance" OR productivity  OR "contextual performance" OR "task performance")  AND  TITLE-ABS-KEY("low-income countr*" OR "middle-income countr*" OR LMIC* OR "developing countr*" OR "resource-constrained"  OR Africa OR Asia  OR (AFGHANISTAN OR ALBANIA OR ALGERIA OR ANGOLA OR ARGENTINA OR ARMENIA OR BANGLADESH  OR BENIN OR BHUTAN OR BOLIVIA OR BOTSWANA OR BRAZIL OR "BURKINA FASO" OR BURUNDI OR CAMBODIA  OR CAMEROON OR "CENTRAL AFRICAN REPUBLIC" OR CHAD OR CHINA OR COLOMBIA OR COMOROS OR CONGO  OR "COTE D'IVOIRE" OR CUBA OR DJIBOUTI OR "DOMINICAN REPUBLIC" OR ECUADOR OR EGYPT OR "EL SALVADOR"  OR ETHIOPIA OR FIJI OR GABON OR GAMBIA OR GHANA OR GUATEMALA OR GUINEA OR HAITI OR HONDURAS  OR INDIA OR INDONESIA OR IRAN OR IRAQ OR JAMAICA OR JORDAN OR KAZAKHSTAN OR KENYA OR KIRIBATI  OR KYRGYZSTAN OR LAOS OR LEBANON OR LESOTHO OR LIBERIA OR MADAGASCAR OR MALAWI OR MALAYSIA  OR MALDIVES OR MALI OR MAURITANIA OR MAURITIUS OR MEXICO OR MOLDOVA OR MONGOLIA OR MOROCCO  OR MOZAMBIQUE OR MYANMAR OR NAMIBIA OR NEPAL OR NICARAGUA OR NIGER OR NIGERIA OR PAKISTAN  OR "PAPUA NEW GUINEA" OR PARAGUAY OR PERU OR PHILIPPINES OR RWANDA OR "SAO TOME" OR SENEGAL  OR "SIERRA LEONE" OR "SOLOMON ISLANDS" OR SOMALIA OR "SOUTH AFRICA" OR "SOUTH SUDAN" OR "SRI LANKA"  OR SUDAN OR SURINAME OR SYRIA OR TAJIKISTAN OR TANZANIA OR THAILAND OR "TIMOR-LESTE"  OR TOGO OR TUNISIA OR TURKEY OR UGANDA OR UKRAINE OR UZBEKISTAN OR VANUATU OR VENEZUELA  OR VIETNAM OR YEMEN OR ZAMBIA OR ZIMBABWE)) |
