# Supplementary material for: Unpacking the Role of Financial Incentives in the Motivation and Individual Performance of Nurses in LMICs: A Scoping Review Protocol
Source: Public Health Chall. 2026 Oct 11;5(4):e70404. doi: 10.1002/puh2.70404 (PMC13654303; doi:10.1002/puh2.70404)
Supplement: Supplementary file 2 [file PUH2-5-e70404-s002.docx]

**Supplementary 3 : Grey literature data extraction**

| General information | | | | | | FIS | | | | | | | | | | Motivation | Individual  Performance |
| --- | --- | --- | --- | --- | --- | --- | --- | --- | --- | --- | --- | --- | --- | --- | --- | --- | --- |
|  |  |  |  |  |  |  |  |  |  |  |  |  |  |  |  | (definition, Theoretical Framework, tool, impacts) | |
| Document ID | Title | Author(s)/  Organization(s) | Year | Document type | lang | Country and geographic location (urban/rural) | Setting (hospital, primary care, community) | FIS alone vs part of a broader programme | Funder | FIS design & Implementation | Type of financial incentive (performance pay, allowance, bonus, etc.) | Incentive amount and relative proportion to base salary | Basis for Payment / Formula | Payment frequency and regularity | Bundled Interventions |  |  |
|  |  |  |  |  |  |  |  |  |  |  |  |  |  |  |  |  |  |
|  |  |  |  |  |  |  |  |  |  |  |  |  |  |  |  |  |  |
