# Supplementary material for: Unpacking the Role of Financial Incentives in the Motivation and Individual Performance of Nurses in LMICs: A Scoping Review Protocol
Source: Public Health Chall. 2026 Oct 11;5(4):e70404. doi: 10.1002/puh2.70404 (PMC13654303; doi:10.1002/puh2.70404)
Supplement: Supplementary file 3 [file PUH2-5-e70404-s003.docx]

| **Data extraction form** | | | | | | | | | | | | | | | | | | | | | | | |
| --- | --- | --- | --- | --- | --- | --- | --- | --- | --- | --- | --- | --- | --- | --- | --- | --- | --- | --- | --- | --- | --- | --- | --- |
| **General Study Information** | | | | | | | **FIS Design and Implementation** | | | | | | | | | | **Motivation and Individual Performance Measurement** | | | | | | |
| Author(s) & Year, | Country and geographic location (urban/rural) | Methods/Study design | Setting (hospital, primary care, community) | Study objectives | Duration of study / follow-up period | Sample size | FIS design | Incentive amount and relative proportion to base salary | Other recipients beyond nurses | Basis for Payment / Formula | Payment frequency and regularity | Type of financial incentive (performance pay, allowance, bonus, etc.) | Demographic characteristics (age, gender, experience) | Type of nurses (registered, licensed practical, nurse practitioners) | Use of Funds (facility, team or individual) | Bundled Interventions (Capacity building,Supervision, Managerial and governance arrangements, Non-financial incentives) | **Definition of Motivation** | **Motivation' Theoretical Framework** | **Measurement Tool (Motivation)** | **Definition of Individual Performance** | **Individual Performance Indicators** | **Measurement Tool (Individual Performance)** | **Individual Performance' Theoretical Framework** |
|  |  |  |  |  |  |  |  |  |  |  |  |  |  |  |  |  |  |  |  |  |  |  |  |
|  |  |  |  |  |  |  |  |  |  |  |  |  |  |  |  |  |  |  |  |  |  |  |  |
|  |  |  |  |  |  |  |  |  |  |  |  |  |  |  |  |  |  |  |  |  |  |  |  |
|  |  |  |  |  |  |  |  |  |  |  |  |  |  |  |  |  |  |  |  |  |  |  |  |
|  |  |  |  |  |  |  |  |  |  |  |  |  |  |  |  |  |  |  |  |  |  |  |  |
|  |  |  |  |  |  |  |  |  |  |  |  |  |  |  |  |  |  |  |  |  |  |  |  |
|  |  |  |  |  |  |  |  |  |  |  |  |  |  |  |  |  |  |  |  |  |  |  |  |
|  |  |  |  |  |  |  |  |  |  |  |  |  |  |  |  |  |  |  |  |  |  |  |  |
|  |  |  |  |  |  |  |  |  |  |  |  |  |  |  |  |  |  |  |  |  |  |  |  |
|  |  |  |  |  |  |  |  |  |  |  |  |  |  |  |  |  |  |  |  |  |  |  |  |
|  |  |  |  |  |  |  |  |  |  |  |  |  |  |  |  |  |  |  |  |  |  |  |  |
|  |  |  |  |  |  |  |  |  |  |  |  |  |  |  |  |  |  |  |  |  |  |  |  |
|  |  |  |  |  |  |  |  |  |  |  |  |  |  |  |  |  |  |  |  |  |  |  |  |

**Supplementary file 2 : DATA EXTRACTION FORM**
