# Supplementary material for: Unpacking the Role of Financial Incentives in the Motivation and Individual Performance of Nurses in LMICs: A Scoping Review Protocol
Source: Public Health Chall. 2026 Oct 11;5(4):e70404. doi: 10.1002/puh2.70404 (PMC13654303; doi:10.1002/puh2.70404)
Supplement: Supplementary file 5 [file PUH2-5-e70404-s005.docx]

| **Authors (Year) / Study ID** | **Define Sub-groups and Studies** | **Narrative Commentary by Study** | **Sub-group Synthesis** | **Overall Synthesis** | **Gaps and limitations** |
| --- | --- | --- | --- | --- | --- |
|  |  |  |  |  |  |
|  |  |  |  |  |  |
|  |  |  |  |  |  |
|  |  |  |  |  |  |
|  |  |  |  |  |  |
|  |  |  |  |  |  |
|  |  |  |  |  |  |
|  |  |  |  |  |  |
|  |  |  |  |  |  |
|  |  |  |  |  |  |
|  |  |  |  |  |  |
|  |  |  |  |  |  |
|  |  |  |  |  |  |
|  |  |  |  |  |  |
|  |  |  |  |  |  |
|  |  |  |  |  |  |
|  |  |  |  |  |  |

**Supplementary file 5 : Textual narrative synthesis**
